# Investigating the early diagnostic value of popliteal artery wall shear stress in lower extremity arterial disease in type 2 diabetes patients using color Doppler ultrasound combined with WSS quantitative analysis

**DOI:** 10.1186/s12938-026-01539-0

**Published:** 2026-02-26

**Authors:** Yuxin Zhao, He Wang, Junyi Gu, Yuqing Sun, Bo Zhang

**Affiliations:** Department of Ultrasound in Medicine, Shanghai East Hospital, School of Medicine, Tongji University, Shanghai, 200120 China

**Keywords:** Type 2 diabetes mellitus, Lower extremity arterial disease, Wall shear stress, Color Doppler ultrasound, Popliteal artery, Atherosclerosis

## Abstract

**Background:**

The global increase in type 2 diabetes mellitus (T2DM) has contributed to a growing burden of lower extremity arterial disease (LEAD), often detected at advanced stages due to limitations in current diagnostic methods. While the ankle-brachial index (ABI) is widely used, it provides no structural information; advanced imaging such as DSA, CTA, or MRA involves radiation, contrast agents, or high cost. Color Doppler ultrasound (DUS) offers a non-invasive and economical alternative for hemodynamic assessment. This study proposes an integrated approach combining DUS with custom MATLAB-based software to quantify wall shear stress (WSS) in the popliteal artery, aiming to establish WSS as a potential early marker of LEAD in T2DM patients.

**Results:**

In a study comparing T2DM patients to controls, T2DM groups showed significantly altered blood pressure, blood lipids, and blood viscosity, along with reduced WSS values, indicating advanced arterial damage. Specifically, WSS was lower in T2DM groups with normal and thickening IMT and those with plaque formation compared to controls. The optimal WSS cutoff for predicting LEAD was 1.82 dyne/cm^2^, with a sensitivity of 68% and specificity of 83%. WSS negatively correlated with factors like age and disease duration, and positively with peak systolic velocity (PSV).

**Conclusions:**

Non-invasive WSS measurement using DUS provides a valuable diagnostic tool for early LEAD detection in T2DM patients. Reduced WSS in the popliteal artery is a predictive marker of disease onset, offering potential for earlier intervention and better management of LEAD, ultimately improving patient outcomes.

## Background

The worldwide explosion of type-2 diabetes mellitus (T2DM), driven by rapid socioeconomic transition and sedentary lifestyles, has propelled lower-extremity arterial disease (LEAD) to epidemic proportions as its most feared vascular complication [1]. Once established, advanced LEAD is synonymous with tissue necrosis, major amputation, and markedly reduced survival, while the escalating costs of multilevel endovascular and surgical revascularization impose an additional societal burden [2]. Pathologically, LEAD progresses through segmental, yet hierarchically selective, atherosclerotic involvement; hemodynamically significant stenoses cluster in the conductance arteries of the leg, with the popliteal artery acting as the critical “calf-perfusion pump” [3]. Tragically, the earliest phases of the disease are clinically silent or dismissed as trivial limb coldness, delaying diagnosis until irreversible ischemia has developed. This asymptomatic window underscores the urgent need for accurate, low-cost screening tools capable of intercepting LEAD before catastrophic tissue loss and resource-intensive interventions become inevitable [2].

At present, LEAD is diagnosed principally with digital subtraction angiography (DSA), CT angiography (CTA), magnetic resonance angiography (MRA), and color doppler ultrasound (DUS) [4]. DSA remains the invasive reference standard, requiring arterial catheterization, whereas CTA and MRA mandate intravenous iodinated or gadolinium-based contrast. All three modalities carry risks of contrast agent allergies, with CTA and DSA additionally exposing patients to ionizing radiation. In contrast, DUS is non-invasive, safe, and inexpensive, providing not only high-resolution structural images but also real-time hemodynamic flow visualization [5, 6].

Hemodynamic characterization is pivotal because the atherosclerotic plaque that defines LEAD is initiated and propelled by aberrant wall shear stress (WSS)—the tangential drag exerted on the endothelium [6]. Sustained low WSS promotes a pathological remodeling response in the arterial wall, leading to an increase in the intima-media thickness (IMT), which is defined as the composite thickness of the innermost two layers of the vessel wall. This makes IMT a well-established structural biomarker for early, subclinical atherosclerotic disease [7].

Among the available techniques, Phase-contrast MRI represents the current reference standard for in vivo hemodynamic analysis, as it captures 3D velocity vectors to permit direct WSS computation without geometric assumptions. However, its clinical translation is constrained by cost and accessibility [8]. DUS, while traditionally indirect, offers unparalleled advantages for longitudinal monitoring: its real-time capability, low cost, and widespread availability are ideal for large-scale diabetic cohorts. Technically, WSS quantification using DUS relies on two primary approaches. Vector Flow Imaging (VFI) provides an innovative, angle-independent assessment of blood flow by resolving two-dimensional velocity vectors. This is achieved through techniques such as transverse oscillation and plane wave imaging, which calculate true flow vectors without the need for angle correction [9]. The method has been successfully applied to directly assess WSS in patients with T2DM, particularly at the carotid artery [10]. While VFI offers a more direct evaluation of complex flow patterns, it typically requires specialized hardware not available in all clinical settings. In contrast, Pulsed-Wave Doppler (PW) is a standard feature on virtually all clinical ultrasound systems. We therefore engineered a custom MATLAB-based WSS analysis software that operates by processing DUS-acquired color Doppler flow imaging (CDFI) and integrating spectral velocity data from PW Doppler to calculate near-wall velocity gradients. This approach was optimally applied to the popliteal artery, which serves as the calf’s primary hemodynamic conduit. Its fixed, straight anatomical course through the adductor hiatus minimizes geometric deviations from ideal flow assumptions, thereby ensuring stable and reliable hemodynamic characterization. This marriage of clinical feasibility and quantitative rigor converts conventional ultrasound into an inexpensive, scalable screening platform for the pre-symptomatic detection and hemodynamic risk stratification of LEAD.

## Results

### Clinical & laboratory results

Clinical and laboratory results for the control group and the T2DM group are summarized in Table 1. Significant differences were observed in SBP, and BMI, with higher values in the T2DM group, and levels of HCT, Fib, TC, TG, LDL-C, FPG, 2 h-PBG, HbA1c, and GA were also significantly higher compared to the control group (*P* < 0.05). There were no significant differences between the groups in terms of Age, DBP, MAP and Cr (*P* > 0.05).

**Table 1:** Clinical and laboratory characteristics in the control group and the T2DM group

Variable	Control group	T2DM group	*P*-value
Age (years)	58.22 ± 10.35	61.56 ± 13.35	0.059
SBP (mmHg)	119.56 ± 12.28	126.13 ± 14.26	< 0.05^*^
DBP (mmHg)	73.01 ± 8.20	79.58 ± 48.46	0.264
MAP (mmHg)	88.53 ± 8.50	95.10 ± 33.37	0.107
BMI (kg/m^2^)	22.91 ± 3.00	25.16 ± 4.37	< 0.05^*^
HCT (%)	38.79 ± 3.04	40.85 ± 4.66	< 0.05^*^
Fib (g/l)	2.77 ± 0.59	3.11 ± 1.02	< 0.05^*^
TC (mmol/l)	3.45 ± 0.93	3.85 ± 1.17	< 0.05^*^
TG (mmol/l)	1.14 ± 0.43	1.59 ± 1.08	< 0.05^*^
HDL-C (mmol/l)	1.47 ± 0.45	1.23 ± 0.56	< 0.05^*^
LDL-C (mmol/l)	2.13 ± 0.77	2.40 ± 1.05	< 0.05^*^
Cr (mmol/l)	68.86 ± 12.63	72.79 ± 23.89	0.193
FPG (mmol/l)	5.39 ± 0.75	8.63 ± 2.79	< 0.05^*^
2 h-PBG (mmol/l)	6.66 ± 0.69	14.40 ± 4.57	< 0.05^*^
HbA1c (%)	5.36 ± 0.57	9.48 ± 2.47	< 0.05^*^
GA (%)	14.76 ± 1.45	25.44 ± 9.88	< 0.05^*^

^*^P < 0.05; **P < 0.01; ***P < *0.001*

Clinical and laboratory results for the control group, T2DM IMT normal group, T2DM IMT thickening group, and T2DM plaque formation group are summarized in Table 2. Compared to the control group, the T2DM IMT normal group showed increased BMI, HCT, TC, TG, FPG, 2 h-PBG, HbA1c, and GA, while age was decreased (*P* < 0.05). The T2DM IMT thickening group, compared to the control group, exhibited increases in BMI, HCT, FPG, 2 h-PBG, HbA1c, and GA, with a decrease in HDL-C (*P* < 0.05). The T2DM plaque formation group, in comparison to the control group, showed increases in age, SBP, BMI, HCT, TG, FPG, 2 h-PBG, HbA1c, and GA, with a decrease in HDL-C (*P* < 0.05). When comparing the T2DM IMT thickening group to the T2DM IMT normal group, there were increases in age and disease duration, with a decrease in TG (*P* < 0.05). Comparing the T2DM with plaque formation group to the T2DM IMT normal group, there were increases in age and disease duration (*P* < 0.05). When comparing the T2DM with plaque formation group to the T2DM IMT thickening group, there was an increase in age (*P* < 0.05). Between the control group, T2DM normal IMT, T2DM increased IMT, and T2DM plaque formation groups, there were no significant differences in DBP, MAP, Fib, LDL-C, Cr across any of the groups (*P* > 0.05).

**Table 2:** Clinical and laboratory characteristics in the control group, T2DM IMT normal group, T2DM IMT thickening group, and T2DM plaque formation group

Variable	Control group	T2DM group		
		IMT normal	IMT thickening	Plaque formation
Age (years)	58.22 ± 10.35^b^	50.04 ± 13.6^c^	61.53 ± 8.83^b^	67.17 ± 12.75^a^
Disease duration (years)	–	8.27 ± 8.10^b^	12.28 ± 7.54^a^	14.35 ± 8.75^a^
SBP (mmHg)	119.56 ± 12.28^b^	124.16 ± 13.54^ab^	124.45 ± 12.46^ab^	127.93 ± 15.06^a^
DBP (mmHg)	73.01 ± 8.20^a^	78.81 ± 8.47^a^	75.51 ± 7.29^a^	82.70 ± 68.77^a^
MAP (mmHg)	88.53 ± 8.50^a^	93.92 ± 9.39^a^	91.82 ± 8.08^a^	97.77 ± 46.78^a^
BMI (kg/m^2^)	22.91 ± 3.00^b^	25.76 ± 5.0^a^	24.80 ± 5.07^a^	25.06 ± 3.46^a^
HCT (%)	38.79 ± 3.04^b^	41.79 ± 5.26^a^	41.12 ± 3.95^a^	41.00 ± 4.64^a^
Fib (g/l)	2.77 ± 0.59^a^	3.03 ± 0.91^a^	3.13 ± 0.98^a^	3.14 ± 1.07^a^
TC (mmol/l)	3.45 ± 0.93^b^	4.06 ± 1.12^a^	3.76 ± 1.22^ab^	3.81 ± 1.15^ab^
TG (mmol/l)	1.14 ± 0.43^c^	1.86 ± 1.02^a^	1.36 ± 0.67^bc^	1.59 ± 1.25^ab^
HDL-C (mmol/l)	1.47 ± 0.45^a^	1.25 ± 0.63^ab^	1.21 ± 0.44^b^	1.23 ± 0.59^b^
LDL-C (mmol/l)	2.13 ± 0.77^a^	2.48 ± 0.98^a^	2.41 ± 1.06^a^	2.35 ± 1.07^a^
Cr (mmol/l)	68.86 ± 12.63^a^	67.45 ± 21.19^a^	69.89 ± 14.74^a^	76.97 ± 28.00^a^
FPG (mmol/l)	5.39 ± 0.75^b^	8.82 ± 2.2.79^a^	8.49 ± 2.39^a^	8.61 ± 2.98^a^
2 h-PBG (mmol/l)	6.66 ± 0.69^b^	14.31 ± 4.38^a^	13.74 ± 3.83^a^	14.81 ± 4.96^a^
HbA1c (%)	5.36 ± 0.57^b^	8.95 ± 2.57^a^	9.53 ± 2.38^a^	9.72 ± 2.42^a^
GA (%)	14.76 ± 1.45^b^	24.61 ± 9.21^a^	25.09 ± 8.16^a^	26.04 ± 10.93^a^

Groups sharing the same superscript letter (a, b, c) indicate no statistically significant difference (*P* > 0.05) for that specific variable. Groups with different superscript letters differ significantly (*P* < 0.05)

### Popliteal artery ultrasound & WSS quantitative analysis results

Popliteal artery ultrasound parameters and quantitative WSS for the control group and the T2DM group are shown in Table 3. Compared to the control group, the T2DM group showed a significant reduction in PSV and WSS (*P* < 0.05). There were no significant differences in R between the two groups (*P *> 0.05).

**Table 3:** Popliteal artery ultrasound and WSS parameters in the control group and the T2DM Group

Variable	Control group	T2DM group	*P*-value
R (mm)	4.56 ± 0.79	4.75 ± 0.89	0.114
PSV (cm/s)	65.35 ± 13.94	51.31 ± 14.81	< 0.05^*^
WSS (dyne/cm^2^)	2.36 ± 0.43	1.81 ± 0.53	< 0.05^*^

^*^P < 0.05; **P < 0.01; ***P < *0.001*

As shown in Table 4, compared to the control group, the T2DM IMT normal group exhibited a significant reduction in WSS (*P* < 0.05). Both PSV and WSS were significantly reduced in the T2DM IMT thickening group compared to the control group (*P* < 0.05). Similarly, the T2DM plaque formation group exhibited significant reductions in PSV and WSS compared to the control group (*P* < 0.05). When comparing the T2DM IMT thickening group to the T2DM IMT normal group, a significant decrease in WSS was observed (*P* < 0.05). The T2DM plaque formation group showed significant reductions in PSV and WSS compared to the T2DM IMT normal group (*P* < 0.05). Additionally, PSV was significantly lower in the T2DM plaque formation group compared to the T2DM IMT thickening group (*P* < 0.05). There were no significant differences in R among the four groups (*P *> 0.05).

**Table 4:** Popliteal artery ultrasound and WSS parameters in the control group, T2DM IMT normal group, T2DM IMT thickening group, and T2DM plaque formation group

Variable	Control group	T2DM group		
		IMT normal	IMT thickening	Plaque formation
R(mm)	4.56 ± 0.79^a^	4.70 ± 0.81^a^	4.86 ± 0.78^a^	4.71 ± 0.97^a^
PSV(cm/s)	65.35 ± 13.94^a^	60.15 ± 12.16^ab^	58.53 ± 13.30^b^	43.03 ± 11.83^c^
WSS(dyne/cm^2^)	2.36 ± 0.43^a^	2.11 ± 0.60^b^	1.81 ± 0.44^c^	1.67 ± 0.48^c^

Groups sharing the same superscript letter (a, b, c) indicate no statistically significant difference (*P* > 0.05) for that specific variable. Groups with different superscript letters differ significantly (*P* < 0.05)

### Popliteal artery WSS predicts the occurrence of LEAD in T2DM patients

**Fig. 1:**
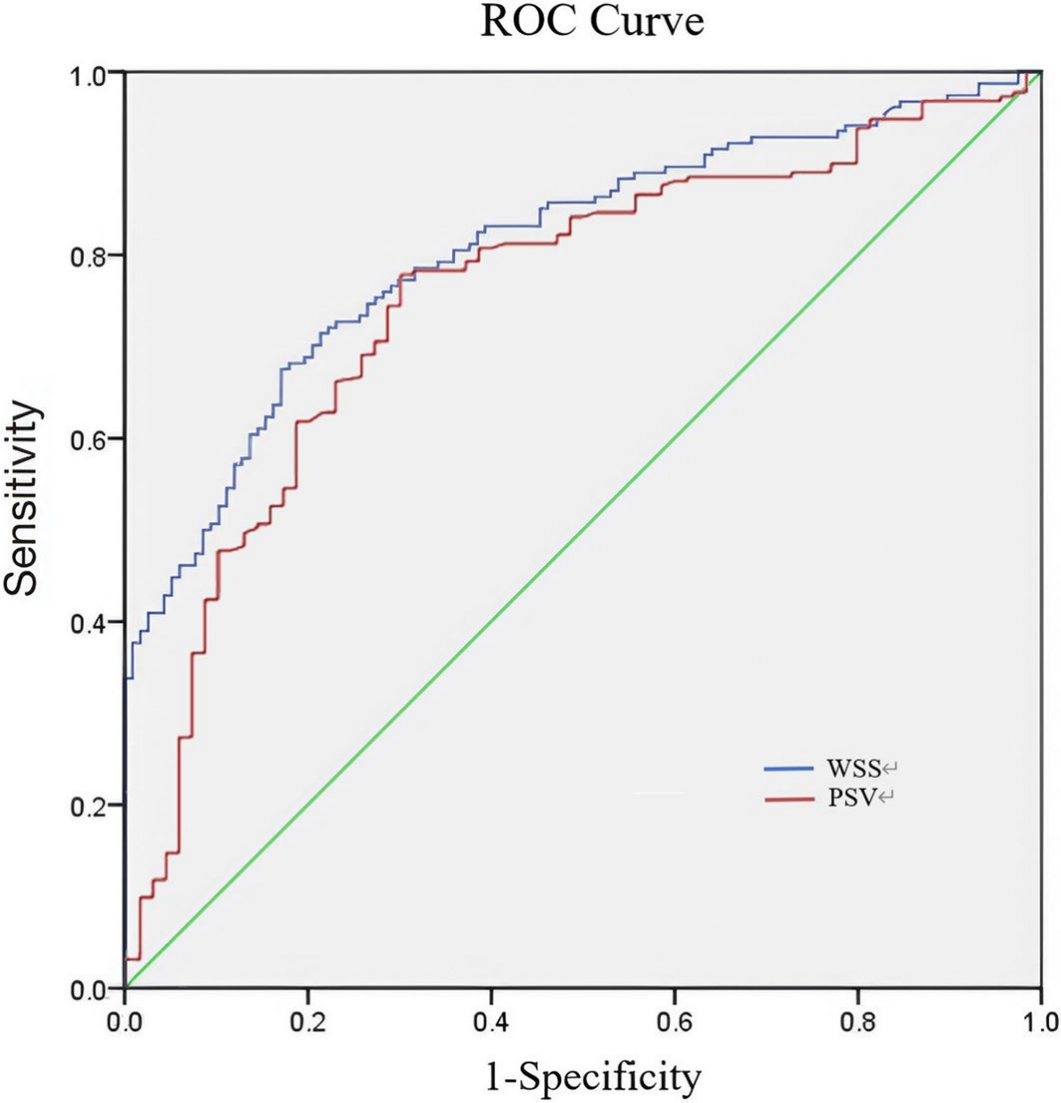
AUC for predicting LEAD in T2DM patients using popliteal artery WSS and PSV

Within the T2DM cohort, participants were classified as positive if either intima-media thickening or plaque was present in the popliteal artery, and as negative when IMT was normal. ROC analysis demonstrated that WSS was the stronger predictor of early LEAD, with an area under the curve (AUC) of 0.81 (95% CI 0.76–0.86), significantly exceeding the AUC of 0.76 (95% CI 0.70–0.83) achieved by PSV (both *P* < 0.001) (Table 5 and Fig. 1).

**Table 5:** AUC for LEAD prediction in T2DM patients through popliteal artery WSS and PSV analysis

Variable	AUC	Standard error	*P*-value	95% CI	
				Lower upper	
WSS(dyne/cm^2^)	0.81	0.026	< 0.001^***^	0.76	0.86
PSV(cm/s)	0.76	0.033	< 0.001^***^	0.70	0.83

^*^P < 0.05; **P < 0.01; ***P < *0.001*

Using the maximum Youden index as the optimization criterion, the best discriminating threshold for popliteal-artery WSS was 1.82 dyne/cm^2^ and for PSV was 61.9 cm/s. At these cut-offs, WSS yielded a specificity of 0.83, substantially higher than the 0.30 achieved by PSV, whereas PSV showed a sensitivity of 0.79 versus 0.68 for WSS. Consequently, WSS provides greater confidence in confirming early-stage LEAD among T2DM patients, whereas PSV is more sensitive but carries a higher false-positive rate (Table 6).

**Table 6:** The optimal cutoff value for predicting LEAD in T2DM patients using popliteal artery WSS and PSV

Variable	Cut-off value	Sensitivity	Specificity	Youden’s index
WSS (dyne/cm^2^)	1.82	0.68	0.83	0.50
PSV (cm/s)	61.90	0.79	0.30	0.49

### Factors correlated with popliteal artery WSS in T2DM group

**Fig. 2:**
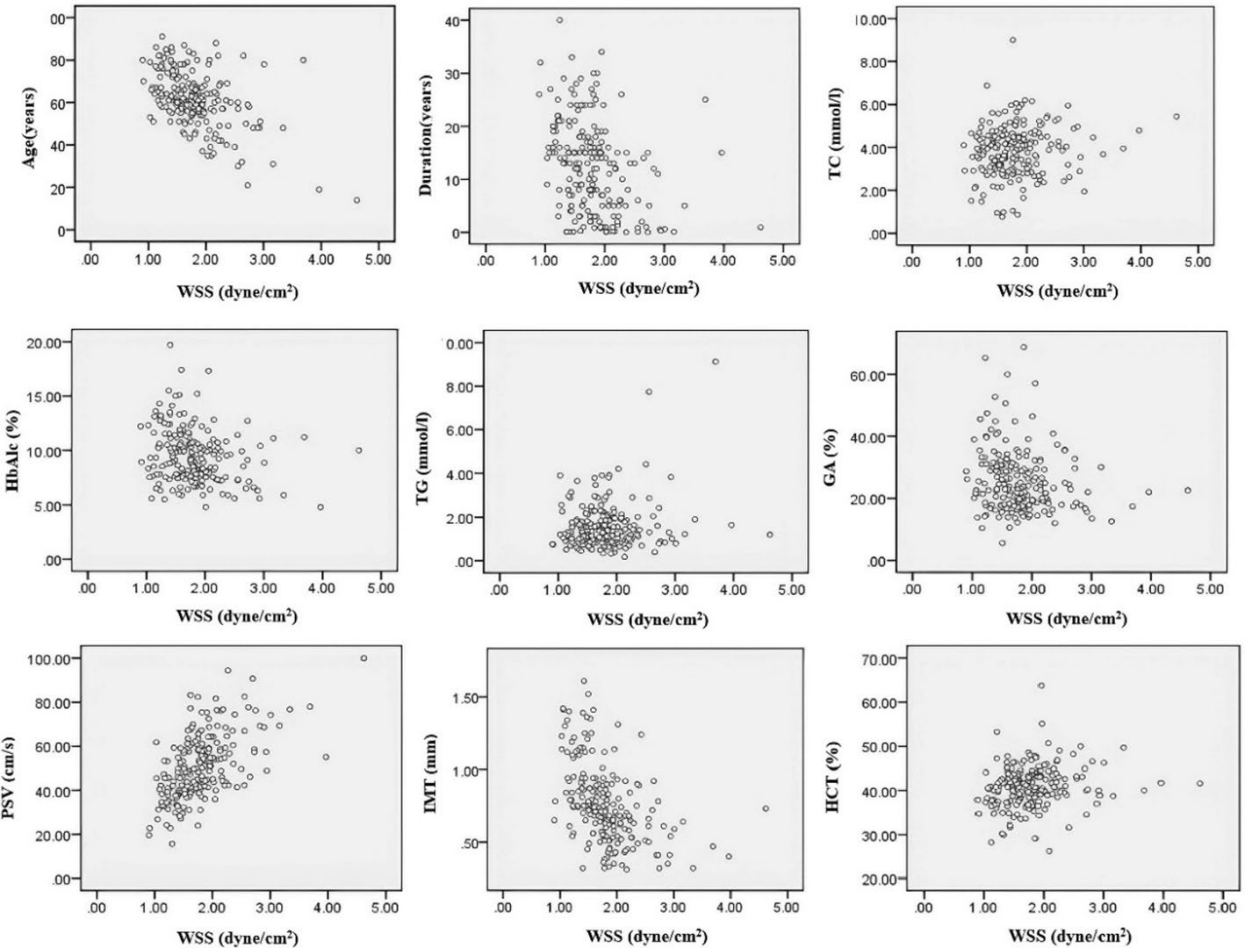
Scatter plot of popliteal artery WSS and related factors in the T2DM group

In the T2DM group, popliteal artery WSS was found to be significantly negatively correlated with age, duration of diabetes, and popliteal artery IMT, and positively correlated with PSV (*P* < 0.001). WSS showed a negative correlation with GA (*P* < 0.01). Additionally, WSS exhibited weak positive correlations with TC, TG, HCT, and a weak negative correlation with HbA1c (*P* < 0.05). There were no significant correlations between WSS and SBP, DBP, MAP, BMI, HDL-C, LDL-C, Cr, Fib, FPG, 2 h-PBG and radius R (P > 0.05) (Table 7 and Fig. 2).

**Table 7:** Analysis of the correlation between different parameters and WSS in T2DM group

Variable		Age	Duration	SBP	DBP	MAP
		(years)	(years)	(mmHg)	(mmHg)	(mmHg)
WSS	r	− 0.50^***^	− 0.34^***^	− 0.10	0.01	0
	P^	< 0.001	< 0.001	0.18	0.84	0.99
Variable		BMI	TC	TG	HDL-C	LDL-C
		(kg/m^2^)	(mmol/l)	(mmol/l)	(mmol/l)	(mmol/l)
WSS	r	0.08	0.14^*^	0.17^*^	− 0.1	0.09
	P^	0.26	0.04	0.01	0.17	0.21
Variable		Cr	HCT	Fib	FPG	PBG
		(mmol/l)	(%)	(%)	(mmol/l)	(mmol/l)
WSS	r	− 0.02	0.18^*^	− 0.09	0.09	0.02
	P^	0.75	0.01	0.23	0.21	0.82
Variable		HbAlc	GA	IMT	R	PSV
		(%)	(%)	(mm)	(mm)	(cm/s)
WSS	r	− 0.21^*^	− 0.18^**^	− 0.53^***^	− 0.04	0.57^***^
	P^	0.01	< 0.01	< 0.001	0.61	< 0.001

r correlation coefficient

^ denotes Pearson correlation analysis

*P < 0.05; **P < 0.01; ***P < 0.001

### Multiple linear regression analysis of popliteal artery WSS in the T2DM group

Using WSS as the dependent variable, and age, duration of disease, TC, TG, HCT, HbA1c, GA, popliteal artery IMT, and PSV as independent risk factors, a multiple linear regression analysis was conducted. The positive correlation coefficient *R* = 0.707 indicates a closely linear relationship and good model fit. WSS is positively correlated with PSV and negatively correlated with age, duration of disease, and popliteal artery IMT, all of which can serve as independent influencing factors affecting WSS (*P* < 0.05) *(*Tables 8 and 9*)*.

**Table 8:** multiple linear regression model

R	R^2^	Adjusted R^2^	Se
0.707a	0.499	0.476	0.387

*a* Predictive variables include age, duration of disease, TC, TG, HCT, HbA1c, GA), popliteal artery IMT, PSV

**Table 9:** multiple linear regression coefficients of variables

Variable	Standardized coefficients	*t*	*P*-value
Age (years)	− 0.246	− 4.125	< 0.05^*^
Duration (years)	− 0.15	− 2.585	< 0.05^*^
TC (mmol/l)	0.028	0.51	0.611
TG (mmol/l)	0.094	1.738	0.084
HCT (%)	− 0.04	− 0.731	0.466
HbA1c (%)	− 0.109	− 1.451	0.148
GA (%)	− 0.004	− 0.048	0.962
IMT (mm)	− 0.23	− 3.959	< 0.05^*^
PSV (cm/s)	0.312	5.076	< 0.05^*^

^*^P < 0.05; **P < 0.01; ***P < *0.001*

## Discussion

The disproportionate burden of LEAD in type 2 diabetes demands a biomarker that can be captured before anatomy is irreversibly altered. By employing a novel DUS-based hemodynamic profiling technique, our study establishes popliteal artery WSS, precisely quantified through our customized MATLAB algorithm that analyzes near-wall velocity gradients from routine clinical Doppler data, as a sensitive hemodynamic biomarker preceding structural atherosclerotic changes in T2DM. By converting standard CDFI data into precise, angle-corrected WSS maps, the technique upgrades conventional morphology-based imaging to a functional hemodynamic screen, permitting detection of the pre-clinical, pre-stenotic phase of LEAD while the vessel lumen is still macroscopically normal.

To evaluate the efficacy of our proposed WSS quantification technique, we compared hemodynamic parameters between the T2DM and control groups. Our quantitative analysis revealed significantly lower PSV and WSS in the T2DM group (*P* < 0.05), suggesting hemodynamic alterations in early diabetes. This finding is mechanistically supported by recent evidence demonstrating that low WSS directly induces endothelial dysfunction via the GRK2/AP-1 signaling pathway, a process further exacerbated by diabetes-specific impairments in endothelial autophagy [11, 12]. Thus, reduced WSS likely acts synergistically with metabolic disturbances to promote early LEAD development. Critically, all T2DM subgroups exhibited reduced WSS versus controls (*P* < 0.05), supporting our technique’s ability to detect incipient atherosclerosis. This sensitivity to disease progression is further evidenced by the significantly lower PSV in the plaque formation group compared to both IMT-normal and IMT-thickening subgroups (*P* < 0.05). Mechanistically, this PSV reduction lowers near-wall velocity gradients, directly leading to WSS depression. In plaque-bearing segments, atherosclerotic lesions disrupt laminar flow through wall irregularities and inflammatory rheological alterations, promoting localized WSS reduction [13]. Although the T2DM plaque formation group showed the lowest mean WSS, the lack of a significant difference versus the IMT-thickening group (*P* > 0.05) likely reflects the complex, non-ideal hemodynamics induced by early plaque, which challenge simplified flow models [14].

*Multivariate regression analysis confirmed the robustness of our findings against confounders*: T2DM subgroup analysis demonstrated that plaque-bearing patients were significantly older, had longer diabetes duration, and higher male predominance than IMT-normal diabetics (*P* < 0.01). Although age-related arterial stiffening and male sex are established confounders [15], multivariate regression confirmed popliteal IMT thickness as an independent determinant of reduced WSS after adjusting for age and sex (β = − 0.23, *P* < 0.05). Critically, we provide multiple lines of evidence to isolate diabetes as the primary driver of WSS reduction: Critically, three lines of evidence establish diabetes-specific WSS reduction: (1) Weak or non-significant correlations between WSS and traditional risk factors (SBP, BMI) contrasted with strong inverse correlations to glycemic markers (HbA1c, GA; both P < 0.01; (2) Progressive WSS decline across diabetic subgroups despite comparable diastolic blood pressure and BMI; (3) Independent prediction by diabetes duration after multivariate adjustment (β = − 0.15, *P* < 0.05). The popliteal artery’s anatomical stability further isolates these effects. Mechanistically, these hemodynamic alterations are driven by a diabetes-specific pathophysiology wherein T2DM intrinsically alters arterial wall mechanics via hyperglycemia-induced collagen cross-linking and AGE accumulation [16]. These changes not only underpin a synergistic interplay between arterial remodeling and accelerated atherosclerosis but also directly promote turbulent flow and reduce WSS [17]. This reduction in WSS, consistent with established principles of plaque initiation, directly affects endothelial cells, thereby significantly influencing the vessel wall’s physiological and pathological states.

Blood viscosity is a complex, multifactorial parameter that is challenging to individualize in clinical or screening settings; therefore, a constant blood viscosity of 3.0cP was used for our WSS calculation [18].The estimated Reynolds numbers (Re ~ 850–1080), based on the vessel diameter and peak systolic velocity in our cohort, confirm laminar flow conditions in the popliteal artery, supporting the validity of the velocity-gradient-based WSS computation under this constant viscosity assumption. Notably, while blood viscosity actually increases in low-shear regions [19], this physiological phenomenon strengthens our finding. Our WSS threshold (≤ 1.82 dyne/cm^2^) identifies such low-shear areas. If we accounted for the expected higher local viscosity, the true WSS would be recalculated upward, meaning the actual WSS in these pathological segments is certainly lower than our 1.82 dyne/cm^2^ estimate. Thus, by employing a fixed viscosity, our findings suggest that this cutoff may represent a conservative estimate, potentially identifying hemodynamically compromised vessels and supporting its possible value in early LEAD screening among T2DM patients.

Crucially, the robustness of our WSS quantification framework is rigorously demonstrated across diverse pathophysiological contexts. Our human studies have confirmed the methodological consistency between CDFI-derived and Hagen-Poiseuille calculated WSS (2.31 ± 0.14 vs 2.98 ± 0.15 dyne/cm^2^, *P* = 0.259) [18]. In rabbit models, this technique proved its predictive power, identifying that a WSS reduction to 1.198 dyne/cm^2^ forecasts fibrous plaque formation (AUC = 0.9283) with a 5-week lead time over IMT thickening, while arterial elasticity was inversely correlated with WSS (r = − 0.68, *P* < 0.001), lagging by 2 weeks [20, 21]. Spatial analysis further consistently localized low-WSS regions to plaque-prone anatomical sites [22]. Most pivotally, experimental studies established a direct mechanistic link, showing that sustained low WSS (< 1.5 dyne/cm^2^ for 72 h) triggers a 3.2-fold elevation in Syk phosphorylation (*P* < 0.01), thereby connecting hemodynamic forces to pro-atherogenic signaling pathways [23]. This extensive reproducibility and adaptability across species and disease stages highlight the technique’s potential as a standardizable tool for both research and future clinical application.

Traditional LEAD diagnostics each offer distinct strengths, as DSA delivers the gold-standard anatomic map preferred for surgical planning, while CTA and MRA excel at delineating complex collateral networks and calcified plaques, and ABI offers a rapid, standardized functional gauge. However, these conventional methods primarily detect advanced disease stages. Notably, ABI and WSS quantification share a common conceptual ground as functional, hemodynamic assessment tools, making their comparison particularly relevant. While ABI remains a valuable tool, its utility is predominantly confined to identifying advanced stages of disease, as an abnormal ABI typically manifests only after the development of hemodynamically significant arterial stenosis (> 50%) [24]. Consequently, its sensitivity for detecting early or moderate LEAD is notably low, which translates into high underdiagnosis rates in asymptomatic T2DM patients, with abnormal ABI detection rates of only 42–49% [25]. Although ABI demonstrates excellent reliability for diagnosing severe LEAD, its accuracy substantially decreases for mild stenoses (76.7%), confirming its ineffectiveness in identifying early-stage disease [26]. Furthermore, clinical risk factors alone are inadequate for identifying patients with reduced ABI, underscoring the need for more sensitive diagnostic tools [27]. As ABI is the most widely established first-line non-invasive test for LEAD in clinical practice, particularly in primary care and diabetic clinics, its documented limitations underscore the need for more sensitive diagnostic tools. To bridge this gap, we derived a popliteal-artery WSS threshold of ≤ 1.82 dyne/cm^2^ (sensitivity 68%, specificity 83%, AUC 0.81) for detecting LEAD in T2DM. Critically, the diagnostic performance of WSS (AUC 0.81; sensitivity 68%, specificity 83%) proved superior to that of PSV (AUC 0.76; sensitivity 0.79, specificity 0.30), whose low specificity limits its clinical utility. The high specificity of WSS makes it a more reliable confirmatory tool. This quantitative WSS metric captures subclinical hemodynamic impairment before either ABI decline or overt symptoms manifest, offering an opportunity for earlier intervention when the disease is still modifiable.

Beyond comparisons with established tools, it is crucial to position our methodology among emerging hemodynamic techniques. Recent VFI studies have successfully quantified carotid artery WSS in T2DM patients, demonstrating significant reductions in parameters like maximum and mean WSS at the common carotid artery and its bifurcation [10, 28]. These reductions were significantly associated with the presence of cardiovascular disease and carotid structural and functional changes, thereby robustly confirming WSS as a valid hemodynamic biomarker and highlighting VFI’s superior capability for direct, angle-independent flow visualization in complex vascular geometries. Our strategy is complementary rather than competitive. In contrast, our PW Doppler-based methodology, while leveraging more universally available clinical hardware, employs a fundamentally different computational approach to derive WSS. Our proprietary algorithm operates not on simplified peak velocity or parabolic flow assumptions, but on the direct computation of near-wall velocity gradients extracted from CDFI data. This pixel-level, gradient-based method provides a more physiologically grounded estimate of WSS than conventional ultrasound estimators, bridging a critical gap between the theoretical completeness of VFI and the practical constraints of clinical screening. Consequently, while VFI may offer a more direct visualization of complex flows, our approach establishes a validated, accessible, and computationally efficient pathway for accurate WSS quantification, making it particularly suited for large-scale screening and longitudinal monitoring of diabetic patients in diverse clinical settings.

WSS values recorded in the popliteal artery (2.36 ± 0.43 dyne/cm^2^ in healthy volunteers) were lower than those typically reported for larger conduit vessels such as the common carotid artery. This divergence reflects both anatomical hemodynamics and methodological constraints. Baseline WSS is intrinsically reduced in peripheral muscular arteries compared with central elastic vessels, a pattern documented by MRI [29] and corroborated by VFI studies demonstrating lower mean WSS in the femoropopliteal versus carotid segments [30]. Technically, our WSS was derived from near-wall velocity gradients in CDFI images, differing from full-flow reconstructions used in VFI [31] or phase-contrast MRI. The spatial resolution and inherent image-processing filters of clinical ultrasound systems may thus yield a conservative estimate of the true near-wall gradient. Additionally, the assumption of a fixed blood viscosity (3.0cP) [18] directly scales the absolute WSS output. These methodological features do not, however, compromise internal validity; the approach furnishes a reproducible hemodynamic metric using universally available ultrasound hardware. Accordingly, the diagnostic threshold of ≤ 1.82 dyne/cm^2^ should be regarded as a technique-specific cut-off. Its discriminatory capacity (AUC = 0.81) within the present cohort—rather than numerical concordance with values from other vascular territories or modalities—underpins its clinical utility.

### Limitations

This study has several limitations that warrant consideration. First, our MATLAB-based WSS quantification depends on unidirectional velocity components, necessitating the assumption of laminar flow parallel to the vessel wall, a constraint not faced by VFI techniques. Although this was mitigated by standardized imaging of straight popliteal segments, operator-dependent variability in image acquisition and delineation remains a critical constraint, potentially introducing systematic bias into WSS computations. Second, the generalizability of our findings may be limited as the study cohort was exclusively of Chinese descent, and ethnic disparities in diabetes progression and LEAD prevalence are well-documented. We recognize that a direct, head-to-head validation of our WSS algorithm against an in-vivo gold standard for hemodynamic quantification (such as phase-contrast MRI) or high-fidelity CFD in the popliteal artery remains a future goal. While we have demonstrated multi-faceted evidence for its validity, benchmarking against these gold standards is the next mandatory step and is planned as part of our ongoing validation protocol.

## Conclusions

In conclusion, this study establishes a novel, non-invasive paradigm for early LEAD detection in T2DM by leveraging quantitative WSS analysis of the popliteal artery via color Doppler ultrasound. Our approach improves on existing assessment methods like DSA, MRA, and CTA by providing a robust diagnostic technique that does not rely on external contrast agents or involve ionizing radiation. While offering superior functional and hemodynamic insight compared to ABI, color Doppler compares favorably in terms of clinical practicality, as it utilizes routine ultrasound equipment, adds minimal time to standard examinations, and enables point-of-care assessment. By combining this quantitative accuracy with operational feasibility, our WSS quantification methodology demonstrates potential as a transformative screening tool for early risk stratification and preventive diabetes care. Therefore, popliteal artery WSS is well positioned to emerge as a reliable hemodynamic indicator for the early diagnosis and prevention of LEAD.

## Methods

### Study participants and grouping

From March 2019 to November 2023, we enrolled T2DM patients from Shanghai East Hospital and healthy volunteers from a health examination center. Participants were divided into a T2DM group and a Control group. The study was approved by the Research Ethics Board of East Hospital, Tongji University (Shanghai, China) [Approval 2017 (No.030)].

1. *T2DM group* A total of 202 patients with T2DM (126 males and 76 females; mean age 61.56 ± 13.35 years) were included. All patients met the 1999 WHO diagnostic criteria for diabetes [32]. Exclusion criteria comprised: type 1 or secondary diabetes, primary hyperlipidemia, hypertension, smoking history, history of lower limb arterial surgery, severe cardiac dysfunction or organic heart disease, renal failure, stroke, malignant tumors, or lower extremity arterial stenosis ≥ 50%. We also excluded patients who had recently received lipid-lowering, anticoagulant, or antiplatelet medications, or had recent adjustments to their glucose-lowering regimen.
  Popliteal artery IMT was measured by ultrasound. Based on IMT measurements, T2DM patients were stratified into three subgroups: (i) IMT-normal (IMT < 1.0 mm; *n* = 48; 24 males, 24 females; mean age 50.04 ± 13.6 years); (ii) IMT-thickening (1.0 ≤ IMT < 1.2 mm; *n* = 55; 38 males, 17 females; mean age 61.53 ± 8.83 years); and (iii) Plaque formation (IMT ≥ 1.2 mm; *n* = 99; 64 males, 35 females; mean age 67.17 ± 12.75 years).
  The IMT threshold of ≥ 1.2 mm for defining popliteal artery plaque was selected based on the distinct pathophysiology of this vascular segment. Evidence indicates that the popliteal artery is inherently more disease-prone and possesses a structurally thicker intima compared to other arterial beds [33]. Furthermore, lower extremity atherosclerosis exhibits immuno-inflammatory characteristics distinct from carotid atherosclerosis, supporting the need for site-specific diagnostic criteria [34]. Given that our T2DM cohort represents a population at high risk for early and accelerated arterial disease, the application of this sensitive threshold is essential for identifying incipient plaque formation, aligning with the study’s objective of early risk stratification [35].
2. *Control group* The control group consisted of 69 healthy volunteers (32 males and 37 females; mean age 58.22 ± 10.35 years). Exclusion criteria for controls included a history of diabetes, hypertension, primary hyperlipidemia, lower limb vascular disease, lower limb surgery, heart failure, organic heart disease, renal failure, stroke, malignant tumors, lower extremity arterial stenosis ≥ 50%, or recent use of any medication.
  We enrolled healthy controls for two sequential objectives: first, to quantify the net hemodynamic burden of T2DM by contrasting global WSS metrics between diabetic and non-diabetic individuals; and second, to furnish a physiologically grounded reference frame that allows us to interpret the stepwise deterioration of WSS across the continuum of subclinical atherosclerosis within the diabetic cohort.

### Clinical data & laboratory findings

Clinical data collected included participant records of gender, age, medical history, and duration of diabetes for the T2DM group. Mean arterial pressure (MAP), body mass index (BMI), systolic blood pressure (SBP), and diastolic blood pressure (DBP) were measured for all subjects in both the control group and the T2DM group. The formula for calculating MAP is: MAP = (SBP + 2 × DBP) / 3. SBP and DBP were manually measured using a mercury sphygmomanometer after a 10 min rest period. All participants fasted for 12 h and had venous blood drawn the following morning to test for glycated hemoglobin (HbA1c), fasting plasma glucose (FPG), two-hour postprandial blood glucose (2 h-PBG), glycated albumin (GA), fibrinogen (Fib), hematocrit (HCT), total cholesterol (TC), low-density lipoprotein cholesterol (LDL-C), triglycerides (TG), high-density lipoprotein cholesterol (HDL-C), and creatinine (Cr).

### Inspection methods

Imaging was performed using a Philips EPIQ7 system (Philips Medical Systems, Andover, MA, USA) with an L11-3 linear array transducer. The transducer’s 128 elements and 0.3 mm pitch underpinned its high spatial resolution, while the Doppler capabilities included a velocity discrimination of 1.5 cm/s at a temporal resolution of 30 Hz. Subjects underwent standardized preparation involving 10 min of supine rest at 24 °C with fully exposed lower limbs, followed by prone positioning with relaxed knee joints. Continuous B-mode and color Doppler imaging captured popliteal artery segments from the adductor hiatus to the anterior tibial artery origin at ≥ 30 frames/s, with triplicate acquisitions of three consecutive cardiac cycles per segment.

For IMT quantification, transverse transducer positioning clearly delineated anterior and posterior arterial walls. At the site of maximal IMT thickness, localized magnification enabled precise measurement of posterior wall IMT and luminal diameter, while longitudinal scanning maintained straight vessel alignment with Doppler beam angles strictly < 60°. Color Doppler parameters included a 2-cm sample box and velocity ranges optimized to prevent aliasing. Additionally, peak systolic velocity (PSV) was recorded using PW Doppler, with 5-s cine loops stored in DICOM format (600 × 800 matrix; 0.085 mm/pixel spacing). The lumen diameter was measured in end-diastole from high-resolution B-mode ultrasound images acquired in the transverse plane. It was defined as the shortest distance between the far-wall blood–intima and near-wall intima–blood interfaces, taken at the site of the greatest vessel caliber where flow variation is minimal. The popliteal artery inner radius (R) was then calculated as R = diameter/2. By placing the calipers at the blood–vessel-wall interfaces, this methodology deliberately excludes plaque volume and specifically quantifies the patent lumen.

### Quantitative WSS analysis

**Fig. 3:**
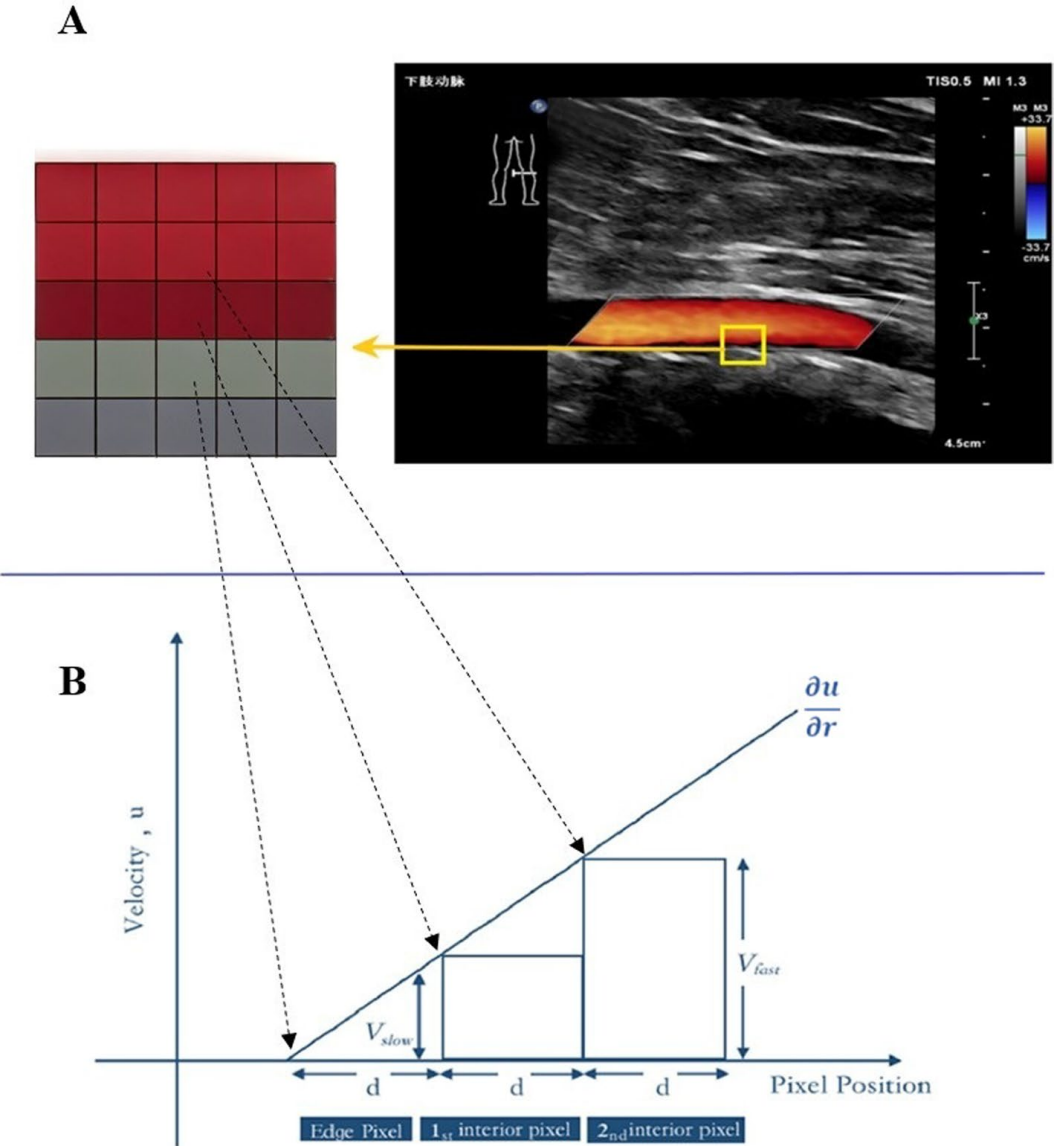
**A** Schematic of the CDFI and the blood-vessel wall interface. The right side shows the CDFI of the popliteal artery. The left side is an enlarged schematic where the upper colored pixels represent near-wall blood flow and the lower grayscale pixels represent the vessel wall. **B** Principle of WSS calculation based on near-wall velocity gradients from CDFI (see Methods, Eq. 3). The lumen boundary (grayscale pixels) has zero velocity. $Vslow$ and $Vfast$ denote the velocities of the first and second adjacent flow pixels perpendicular to the wall, respectively. The velocity gradient is calculated as $\frac{\partial u}{\partial r}=\frac{V{\mathrm{fast}}-\mathrm{Vslow}}{\Delta d}$, where $\Delta d$ is the fixed inter-pixel distance

WSS was quantified using a proprietary, MATLAB^®^-based (The MathWorks Inc., Natick, MA, USA) analysis algorithm developed in-house. The algorithm processes DICOM images from CDFI, which store data as a multi-dimensional matrix (M × N × 3 × frames) representing the RGB color space and temporal sequence. The core WSS computation was then performed using the CDFI data. Crucially, the algorithm leverages the fundamental difference in image encoding: the blood flow signal is encoded in color (RGB), while the surrounding tissue and vessel wall are displayed in grayscale on B-mode imaging. This allows the algorithm to automatically segment and isolate the color Doppler pixels corresponding to blood flow. The color hue and brightness of each pixel correspond to a specific flow velocity and direction, as defined by the ultrasound system’s on-screen color-velocity bar. By referencing this calibrated bar, the algorithm maps each RGB pixel value to an absolute flow velocity, scaled by the preset maximum velocity, thereby reconstructing a two-dimensional, spatially distributed velocity field. This velocity field enables the direct computation of near-wall velocity gradients. The algorithm implements a validated computational pipeline that processes image sequences spanning ≥ 3 cardiac cycles to capture complete hemodynamic pulses. This pipeline includes: anisotropic diffusion filtering to suppress speckle noise while preserving vascular boundaries; optical flow analysis to reconstruct velocity vectors from consecutive frames; and spatial gradient computation applied at the vessel wall interface to extract the shear rate [18].

1 $${\tau }_{w}=\frac{4\mu v}{d}$$

where ${\tau }_{w}$ denotes WSS (1 Pa = 10dyne/cm^2^), $\mu$ blood viscosity, $v$ blood flow velocity, and $d$ vessel diameter. Extending this principle, vessel walls were identified as the first continuous echogenic boundary, manually delineated by two independent operators (intraclass correlation coefficient = 0.91) with boundary continuity requiring ≥ 3 adjacent pixels at intensity > 40% of maximum lumen signal. WSS was subsequently quantified via direct velocity-gradient computation:

2 $${\tau }_{w}=\mu {\gamma }_{w}=\mu \frac{\partial u}{\partial r}{|}_{r=wall}$$

with $\mu$ set to 3.0cP under the Newtonian fluid assumption, ${\gamma }_{w}$ the shear rate (s⁻^1^), and $\frac{\partial u}{{\partial {\mathrm{r}}}}$ the radial velocity gradient ($u$ = axial flow velocity; $\mathrm{r}$ = radial coordinate at the wall, $r=wall$). The shear rate ${\gamma }_{w}$ was derived from the color-Doppler pixel data by calculating the velocity gradient between adjacent near-wall pixels (as schematically illustrated in Fig. 3):

3 $${\gamma }_{w}\approx \frac{\partial u}{\partial r}=\frac{V{\mathrm{fast}}-\mathrm{Vslow}}{\Delta d}$$

Here, $Vfast$ and $Vslow$ denote velocities at near-wall pixels (0–0.3 mm from the boundary), $\Delta d$ is the inter-pixel distance (0.085 mm). This method directly converts the spatial velocity information from CDFI into a local shear rate estimate.

**Fig. 4:**
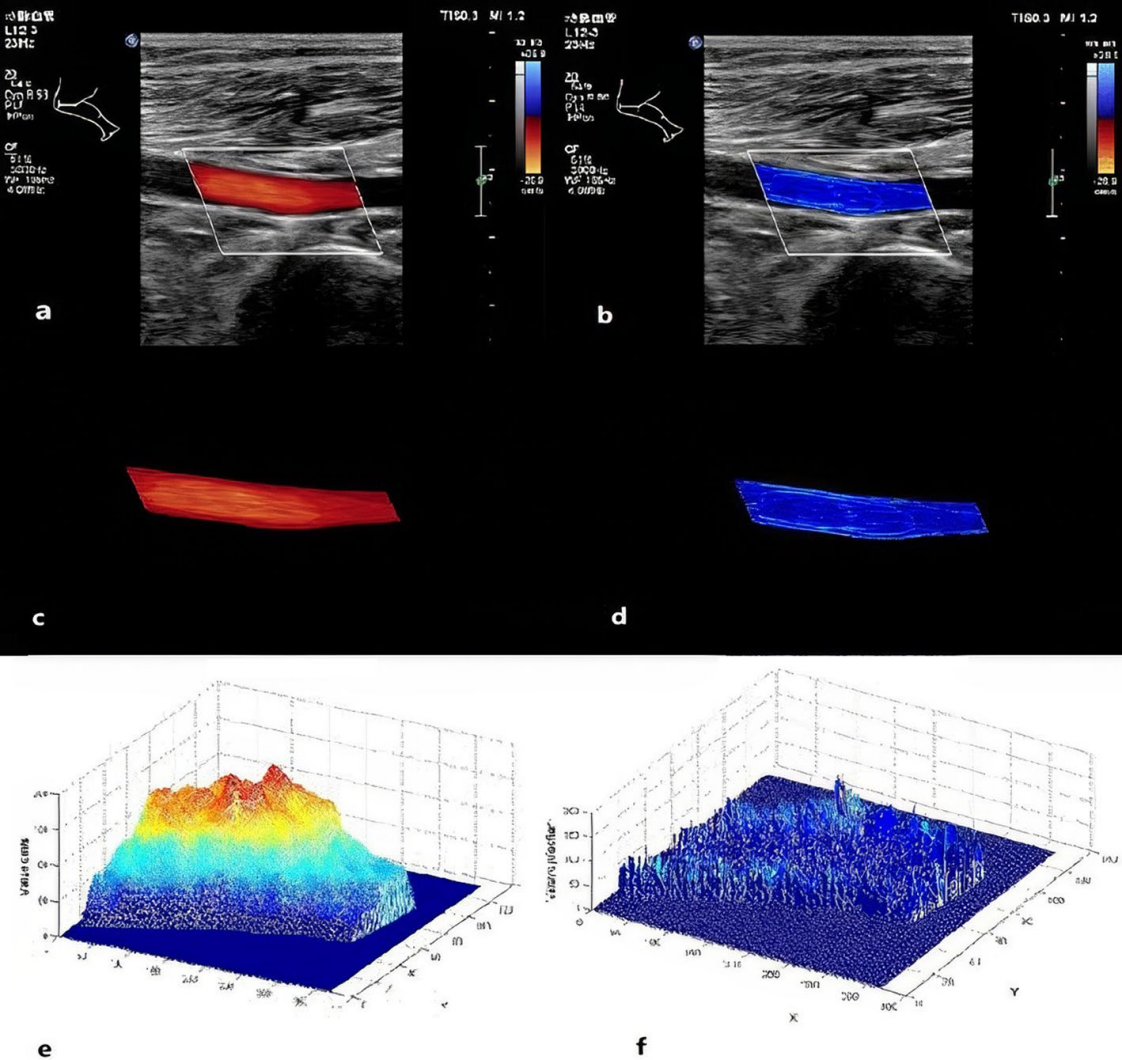
**a** Doppler flow image of the popliteal artery; **b** WSS distribution map fused with the Doppler flow image of the popliteal artery; **c** Segmentation map of the popliteal artery Doppler flow; **d** 2D distribution map of WSS in the popliteal artery; **e** 3D spatial map of Doppler blood flow velocity in the popliteal artery; **f** 3D spatial distribution map of WSS in the popliteal artery

This Hagen–Poiseuille-based pixel-gradient conversion, operating on the automatically segmented flow region from CDFI (based on the color [RGB] versus grayscale signal distinction), enabled pointwise WSS mapping with 100 Hz wall filtering. The pipeline thus generated comprehensive outputs for quantitative assessment (Fig. 4), including: fused WSS-Doppler distribution maps, the corresponding segmentation maps, 2D WSS spatial distributions, and 3D blood flow velocity profiles.

### Theoretical advantages over conventional WSS estimation paradigms

To contextualize our approach, we benchmarked it against conventional ultrasound-based WSS estimators, notably the method by Hoskins et al. which computes wall shear stress as $WSS=\frac{{2\mu V}_{max}}{R}$ (where $\mu$ is the dynamic viscosity of blood, $Vmax$ is the peak velocity recorded at the center of the vessel and $R$ is the vessel radius.) under idealized parabolic flow conditions. While computationally efficient, this approach exhibits systematic overestimation in pathological segments with flow separation or non-parabolic profiles—a limitation confirmed by in-vitro hemodynamic studies [36].Conversely, our near-wall gradient method adheres to the fundamental definition Eq. 2, deriving shear rates Eq. 3 directly from localized velocity gradients. This paradigm eliminates reliance on global parabolic assumptions, demonstrates superior accuracy in complex hemodynamic environments compared to maximum-velocity methods, and enables spatially resolved WSS mapping. The synergy of physical rigor (validated via Hagen-Poiseuille framework, Eq. 2) and computational efficiency positions our approach as clinically optimal for detecting early hemodynamic disturbances.

Computational fluid dynamics resolves the Navier–Stokes equations to model complex three-dimensional hemodynamics, constituting the theoretical gold standard for comprehensive flow analysis [37]. However, its clinical translation faces dual constraints: prohibitive computational demands from high-resolution volumetric meshing, and inherent risks of underestimating wall shear stress due to simplifications of non-periodic flow dynamics—particularly critical in early-stage disease characterization [38]. Our near-wall gradient approach circumvents these limitations by directly implementing the Newtonian viscous principle: quantifying shear stress through localized velocity gradients within the vessel boundary layer. This eliminates CFD’s mesh dependency while preserving hemodynamic fidelity through laminar flow validation, enabling real-time WSS quantification at clinically actionable speeds. The resultant spatially resolved hemodynamic maps thus bridge the gap between theoretical completeness and practical early-detection utility.

### Validation of WSS measurement repeatability

To provide a comprehensive validation of the final WSS output from our entire quantification pipeline, we conducted formal intra- and inter-observer reproducibility analyses. Two independent sonographers (with > 5 and > 10 years of vascular experience) acquired and analyzed images from a randomly selected subset of participants (*n* = 30) on two separate occasions. The intraclass correlation coefficient (ICC) for intra-observer repeatability was 0.91 (95% CI 0.85–0.95), and for inter-observer reproducibility was 0.89 (95% CI 0.82–0.93), indicating excellent reliability of the WSS measurements. Furthermore, the Bland–Altman analysis revealed a mean difference (bias) of 0.08 dyne/cm^2^ with limits of agreement from − 0.35 to 0.51 dyne/cm^2^, demonstrating clinically acceptable variability.

### Statistical analysis

Statistical analysis was performed using SPSS 24.0 (SPSS Inc., Chicago, IL, USA). Continuous variables are presented as mean ± SD, and categorical variables as counts (percentages). The normality of all continuous variables was confirmed via the Shapiro–Wilk test, while homogeneity of variances for parametric comparisons was verified by Levene’s test. Between-group comparisons adhered to the following protocol: For the two-group comparisons (Tables 1 and 3), independent-samples *t* -tests were used for data meeting the assumption of homogeneity of variance, while Welch’s *t*-test was applied when this assumption was violated. For the multi-group comparisons (Tables 2 and 4), one-way analysis of variance (ANOVA) was performed, followed by Tukey’s HSD post-hoc tests. Based on the normality testing, the associations between variables were assessed using Pearson;s correlation coefficient for normally distributed data; otherwise, Spearman’s rank correlation was used (Table 7). The predictive value of popliteal artery WSS and PSV for LEAD in T2DM was evaluated using receiver operating characteristic (ROC) curve analysis (Tables 5 and 6). A multiple linear regression analysis using the Enter method was conducted to identify independent factors influencing WSS, with predictor variables selected based on univariate analyses (*P *< 0.10) and clinical relevance (Tables 8 and 9). Statistical significance was defined as follows: *P* < 0.05 was considered statistically significant, *P* < 0.01 was considered highly significant, and *P* < 0.001 was considered extremely significant.

## Data Availability

The datasets used during the current study are available from the corresponding author on reasonable request.
